# [Retracted] Rab5 regulates the proliferation, migration and invasion of glioma cells via cyclin E

**DOI:** 10.3892/ol.2024.14582

**Published:** 2024-07-23

**Authors:** Zhao Jian, Lianglong Zhang, Liang Jin, Weitu Lan, Wei Zhang, Guiyan Gao

Oncol Lett 20: 1055–1062, 2020; DOI: 10.3892/ol.2020.11660

Following the publication of this paper, it was drawn to the Editor's attention by a concerned reader that certain of the Transwell invasion and migration assay data shown in Fig. 2G on p. 1058 had already appeared in previously published articles written by different authors at different research institutes. Owing to the fact that the contentious data in the above article had already been published prior to its submission to *Oncology Letters*, the Editor has decided that this paper should be retracted from the Journal. The authors were asked for an explanation to account for these concerns, but the Editorial Office did not receive a reply. The Editor apologizes to the readership for any inconvenience caused.

